# A visual identification key utilizing both gestalt and analytic approaches to identification of Carices present in North America (Plantae, Cyperaceae)

**DOI:** 10.3897/BDJ.1.e984

**Published:** 2013-09-16

**Authors:** Timothy Mark Jones

**Affiliations:** † Louisiana State University, Baton Rouge, United States of America

**Keywords:** Visual key, identification, *Carex*, *Cymophyllus*, *Kobresia*, interactive identification, sedges

## Abstract

Images are a critical part of the identification process because they enable direct, immediate and relatively unmediated comparisons between a specimen being identified and one or more reference specimens. The Carices Interactive Visual Identification Key (CIVIK) is a novel tool for identification of North American *Carex* species, the largest vascular plant genus in North America, and two less numerous closely-related genera, *Cymophyllus* and *Kobresia*. CIVIK incorporates 1288 high-resolution tiled image sets that allow users to zoom in to view minute structures that are crucial at times for identification in these genera. Morphological data are derived from the earlier *Carex* Interactive Identification Key (CIIK) which in turn used data from the *Flora of North America* treatments. In this new iteration, images can be viewed in a grid or histogram format, allowing multiple representations of data. In both formats the images are fully zoomable.

## Introduction

The last ten years may be remembered for the rebirth of plant taxonomy and systematics in a new guise, computational biodiversity informatics. For much of the earth, and North America in particular, botanical information that once required substantial effort to acquire is now reliably provided in seconds by such websites as the Global Biodiversity Information Facility (GBIF), Flora of North America, Missouri Botanical Garden's Tropicos, Encyclopedia of Life, United States Plants Database, and emerging regional herbarium networks. Plant biodiversity is now literally at everyone’s fingertips.

### State of the art plant identification systems

Traditional biological identification systems today are of two primary types; analytic and gestalt (K. Thiele, pers. comm. 2013). Two forms of analytic keys commonly used today are dichotomous and interactive matrix-based keys. Both are primarily text-based question systems that can yield static images upon the final determination. Conversely, gestalt keys, use an identifiable image of the organism in question. Similar to what is seen in field guides.

Analytic matrix-based keys are considered to be state of the art today The University Of Queensland 2006 due to their ability to scale up across hundreds of taxa. To use, users select characters to achieve a determination of the unknown taxon using a four-panel informational interface. The information panels often represented are 'characters available', 'characters chosen', 'entities available', and entities discarded'. Within this format, it is possible to insert thumbnail-sized, static images to accompany the text if the taxa numbers are relatively small (< 100). But when taxa numbers are higher (>100), their inclusion results in the information panel becoming too long to be usable, e.g. the Carices used here would require copious scrolling across its many meters of length.

Visual keys borrow from both gestalt and analytic methods. They use character matrices for initial pruning of the image set analytically. After a few characters choices the many hundreds of small images are reduced to a manageable set of bigger images. Now gestalt methods take over as the images become larger and truly informative. With this hybrid of functionality, featuring the best of both gestalt and analysis, a novel identification method is created that can cater to the neophyte as well as the expert.

### Carex, Kobresia, and Cymophyllous: a model for scalability

*Carex* is the largest vascular plant genus in North America (Ball and Reznicek 2002). With two closely related genera, *Kobresia* and *Cymophyllus*, it forms the Carices of North America; all three are members of the family *Cyperaceae*, commonly called sedges but often erroneously referred to as grasses. These three genera share a number of basic morphological characteristics including having linear leaves and a fruit enclosed in a bag-like structure called a perigynium. All have small flowers that lack large, colorful petals and sepals. Plus they share one other important characteristic: they are difficult to identify. Nevertheless, they are morphologically distinct and relatively easily recognizable as a group.

### The new visual key

The data used in this project are primarily derived from an interactive identification program to *Carex* that has been online since 2006 at both Utah State University and Louisiana State University (http://www.herbarium.lsu.edu/keys/carex/carex.html). During this time it has been consistently revised and is currently in version 21. (Suppl. materials 3, 4). Web statistics have been tracked from 2007. Data show that numerous individuals worldwide, government agencies, students in classrooms, and participants in identification workshops have repeatedly used the keys. Many users have graciously suggested revisions and clarifications that have increased their usability and performance. The key presented here reflects contributions from several individuals, innumerable field trips, and countless hours in herbaria both identifying and imaging specimens. It is only with such collaboration and effort that an image key to such a large genus can be created.

### Goals

My goal in this project was to create an easy to use identification resource that maximized the value of high resolution images while enabling users to explore the distribution of morphological diversity within the genera. Query-able images. For example, to answer questions such as: how are species with trigonous achenes geographically distributed across Canada by province or territory? How common are species with two-sided achenes in species with leaf blades more than 10 mm wide? These sorts of hypotheses are easily answered in histogram mode Fig. 4. Because for the first time, side-by-side image comparisons are possible across species permitting comparative examination and discrimination among closely-related members of any complex, of which there are many, within the Carices. CIVIK is seen here: http://www.herbarium2.lsu.edu/aba/

## Development of visual identification tool

### Study area description

This key is designed for use in North America, including Mexico. The original descriptive data was derived from Flora of North America (Ball and Reznicek 2002) and (Mackenzie 1940). My images come from fieldwork focused in eastern North America while other individuals have contributed images from other locations across North America.

### Design description

**1. IMAGES**

**1.1. Contributors**

Steve Matson and Tony Reznicek both sent a DVD copy of their *Carex* field images. Lowell Urbatsch contributed his teaching-microscopy-images (http://www.herbarium.lsu.edu/keys/eee/b52.html). My images were collected from many field sites primarily in the north-eastern United States. The New York Botanical Garden Press granted the use of the plates of both North American Cariceae volumes (Mackenzie 1940[Bibr B112760]). The remaining images were found on the World Wide Web (WWW) and their owners (Forest Starr, Kim Starr, Nhy Nyugen, Ann Debolt) contacted by email to request permission for their use. The remaining image contributor, Robert Mohlenbrock, had made the image used here available on http://www.plants.usda.gov/ so it could be used without seeking permission.

**1.2. Processing of images**

To manage the large image numbers (e.g., Matson hundreds of images; Jones, many thousands), each set of images from each owner was segregated on a local drive. Predictably, across this many image contributors, naming conventions differed greatly, thus significant renaming of image files was required. The basic convention used was to include the taxon name, type of image, and the author in the file name. Another issue of note was the fact that many of these images had been prepared for delivery via the WWW, and had been re-sized. Larger file sizes were selected for inclusion while those that were originally designed as thumbnails were not used. Rarely, older images that were scanned from slides were either cropped or otherwise manipulated with Photoshop CS 3. Lastly, rotation of images for appropriate orientation was also often required.

**1.2.1 Image sizes**

Image sizes are variable and range from 40 K to over 13 MB. Line drawings and most images by Jones are at 2848 × 4288 with a maximal bit depth of 24. Matson's images were more variable as some images had been prepared for web use. They range from 2592 × 3888 to 550 × 689 with variable bit depths. Other contributed images are of intermediate sizes.

**1.3. Imaging of Mackenzie's plates**

New York Botanical Garden Press gave permission to image the plates in K. K. Mackenzie's two volume treatment of Carices of North America (Mackenzie 1940) for use in this project. All plates were imaged with a traditional copy stand, using a Nikon 300D camera with a 1:1 macro lens, and two halogen desk lamps for illumination using JPEG format. All images required batch-processing in Photoshop CS3 for color and a minor defect in skew. Additionally, to limit total file size of the project, the images were reduced to approximately one megabyte from three megabytes by resizing.

**2. DATA FOR CXML CREATION**

**2.1. Primary data via export**

**Figure 1.**
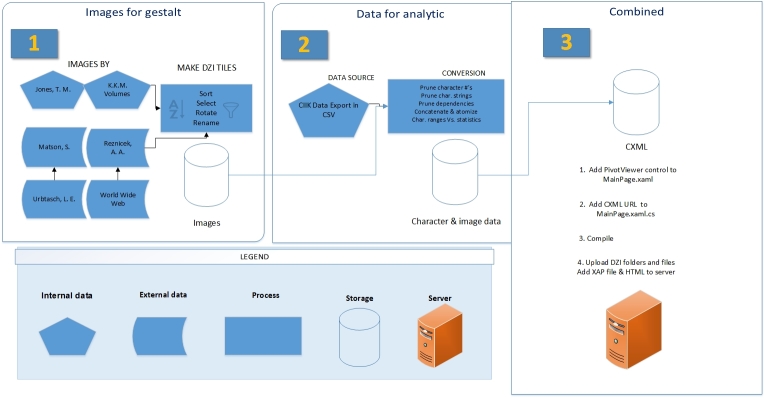
Workflow of project

The dataset was derived from an export of CIIK (http://www.herbarium.lsu.edu/keys/carex/carex.html) in comma separated values (CSV) from LUCID 3.4 Identification Software (The University Of Queensland 2006). These data were the template for the new secondary dataset (Fig. 1). The exported data were imported into Excel 2010 and the Excel PivotViewer plug-in generated the Commerce eXtensible Markup Language (CXML) version of the data (Suppl. material 1). This plugin has since been deprecated in favor of a command line tool, Pauthor (Microsoft 2010a, Microsoft 2010b).

**2.2. Dependent software**

.NET Framework (Microsoft 2007)

Visual Studio 2010 / 2012

Silverlight 4 Tools for Visual Studio 2010

Silverlight Software Development Kit (SDK)

Silverlight 4 Toolkit

PivotViewer SDK

**2.3. Interface considerations in a micro-ontology**

**Figure 2.**
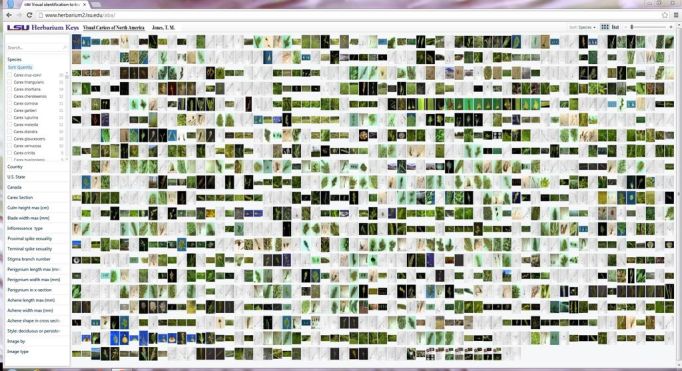
The Visual Carices of North America upon instantiation in default grid setting.

In Pivot Viewer with the Silverlight 4 format, the characters and states (C&S) are located in the searchable information pane on left, with the displayable information pane on right. This left pane is of a fixed width, lacking word-wrapping functions (Fig. 2). If all C&S information data mined were used, extensive scrolling would be required and thereby reduce the usability of the key. For this reason, long text strings in the C&S were edited for brevity. A 'less is more' approach was taken, with C&S being restricted to those that would be appropriate in an ontology.

**2.4. Clustering issues in the graphical mode require a “normalization character state”**

*Visual keys require a normalization character state; or the image numbers must be standardized for graphical display*

If image numbers between species are not consistent, a representative or semantic image is required. This leading image permits true one-to-one comparisons over any number of taxa. Without it, accurate representations of the data would be obscured due to clustering. For this reason, only those taxa with a line drawing are presented here to allow for a one-to-one comparison across taxa. It was done early in development as a work-around to the differing number of images per taxon problem. Later unpublished works of this type deal with this issue in multiple ways (see 'Additional information').

To use this normalization feature, select ‘Image by’ at the base of the left information pane, then select ‘Mackenzie, K. K.’ from the information panel. Now, only grey scale images are used in a portrait format with an attention to the aspect ratio. All images are presented in the same fashion and uniformity in a grey scale that is easy to visually interpret. This ad-hoc commitment to Mackenzie's species list was done for this reason.

**2.5. Data and images together**

Images were added in small batches in a new Excel file. Character data were copy-pasted from the secondary spreadsheet to the third instance of Excel to form the final building file across multiple monitors.

**2.6. Tertiary data**

**Figure 3.**
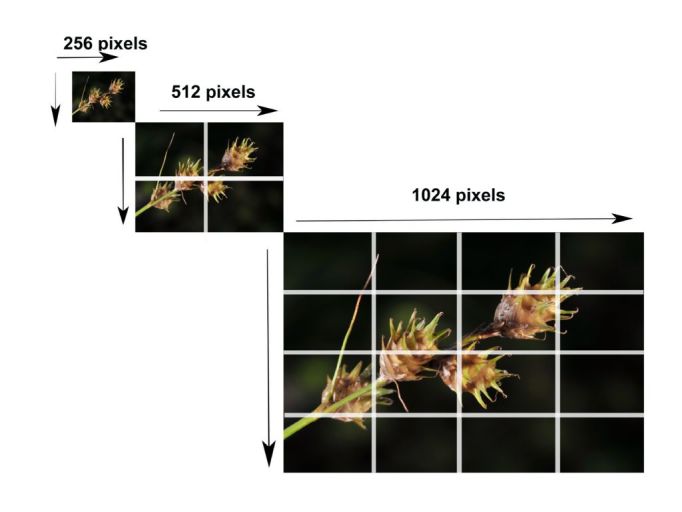
Tiled image set illustrating the change in file size as well as number of images by creating a geometric series of images

The completed third spreadsheet is now run using the 'New collection tool' by selecting its icon in the ribbon panel of Excel. It generates two primary products; image tiles in numerous folders and a CXML file (Suppl. material 1). The control leverages Deepzoom technology (Microsoft 2008) to create a deep zoom image library (DZI) and deep zoom collection files (DZC) like those seen on Google or Bing maps (Fig. 3). This geometric series of images supports the zoom-ability of images. As the user zooms in, things get geometrically resolved without the penalty associated with a large image download. As users pan through a collection, they can see only what they desire.

**2.6.1 Issues completing tertiary data for image tiles and CXML**

Hardware and software issues were experienced at all stages. Testing revealed that while tiling a few hundred high resolution images with PivotViewer is manageable, using over a thousand high-resolution images made Excel unstable. Memory allocation as well as the processor spiking issues - limited development time and resulted in extended periods of waiting for test builds overnight or on a build across many days. The creation of the image tiles is best attempted with a state-of-the-art computer with a solid state drive. CIVIK total tile-set and cxml build-time was approximately 12 hours for the final presented build (Fig. 4).

**3. Deployable image tiles sizes**

The DZI files are nearly four gigabytes in file size and comprise over 250,000 image-tile files in over 18,000 folders with an associated CXML of 3.3 megabytes in size. A Silverlight application package (XAP) file is also required to drive the application.

**4. Compile with Visual Studio**

To compile with Visual Studio, open a new instance of a Silverlight application for the web in Visual Studio. Now add the references to PivotViewer on the main Extensible Application Markup Language (XAML) page in UserControl. Then add the URL to the CXML file to the XAML.CS code behind file. Then, build or compile the deployment package for placement on the server.

**4.1 XAML and XAML.CS Code behind Files**

See 'software technical features'

**5. Deploy to web server**

Ensure that the following Multipurpose Internet Mail Extensions (MIME) types are configured on server; significant development time was lost due to one of these settings not being in place.

• CXML - text/xml

• DZC - text/xml

• DZI - text/xml

**6. History of Use**

CIVIKhas been tracked via Google Analytics with the other later works of visual types. These combined works reveal that 13,933 visits occurred from 116 countries in 2464 cities over a three year period. An average dwell time of two minutes across the three works of type is seen here. (See Additional information and Suppl. material 6).

**7. Considerations and discussion**

While Silverlight is ideal for this data format, it will be deprecated (see http://support.microsoft.com/gp/lifean45) as no future versions are scheduled for release. It will, however, be supported for ten years which will aid future works of this kind. Thankfully, HTML 5 versions are also now available for PivotViewer that enable the CXML format across all devices in a device agnostic fashion. This cross platform capability is exciting as it does not require the Silverlight runtime, so phone and tablets are enabled as well with HTML 5. HTML 5 versions have one other important advantage - a Google translate function is easily added in minutes to over 70 languages (see http://translate.google.com/about/). Opening the door to future iterations of high-resolution images supported by text that is translatable.

### Funding

SLouisiana State University

## Geographic coverage

**Description:** The identification key can be used for species occurring in United States, Canada, and Mexico. Several species have a much wider distribution, hence the key has some value in other regions as well.

**Coordinates:** 90 and 15 Latitude; -180 and -45 Longitude.

## Taxonomic coverage

**Table:** 

Scientific Name	Common Name	Rank
Carex	sedge	genus
Kobresia	sedge	genus
Cymophyllus	sedge	genus
Carex abrupta Mack.	abruptbeak sedge	species
Carex abscondita Mack.	thicket sedge	species
Carex adusta Boott	lesser brown sedge	species
Carex aestivalis M.A. Curtis ex A. Gray	summer sedge	species
Carex aggregata Mack.	glomerate sedge	species
Carex alata Torr.	broadwing sedge	species
Carex albicans Willd. ex Spreng.	whitetinge sedge	species
Carex albonigra Mack.	blackandwhite sedge	species
Carex albursina E. Sheld.	white bear sedge	species
Carex alligata Boott	Hawai'i sedge	species
Carex alma L.H. Bailey	sturdy sedge	species
Carex alopecoidea Tuck.	Foxtail sedge	species
Carex amphibola Steud.	eastern narrowleaf sedge	species
Carex amplectens Mack.	claspbract sedge	species
Carex amplifolia Boott	bigleaf sedge	species
Carex annectens (E.P. Bicknell) E.P. Bicknell	yellowfruit sedge	species
Carex anthoxanthea J. Presl & C. Presl	grassyslope arctic sedge	species
Carex aperta Boott	Columbian sedge	species
Carex aquatilis Wahlenb.	water sedge	species
Carex arapahoensis Clokey	Arapaho sedge	species
Carex arcta Boott	northern cluster sedge	species
Carex arctata Boott	drooping woodland sedge	species
Carex arenaria L.	sand sedge	species
Carex arkansana (L.H. Bailey) L.H. Bailey	Arkansas sedge	species
Carex assiniboinensis W. Boott	Assiniboia sedge	species
Carex atherodes Spreng.	wheat sedge	species
Carex athrostachya Olney	slenderbeak sedge	species
Carex atlantica L. H. Bailey	prickly bog sedge	species
Carex atrata L.	black scale sedge	species
Carex atratiformis Britton	scrabrous black sedge	species
Carex atrofusca Schkuhr	darkbrown sedge	species
Carex atrosquama Mack.	lesser blackscale sedge	species
Carex aurea Nutt.	golden sedge	species
Carex austrina Mack.	southern sedge	species
Carex austrocaroliniana L.H. Bailey	tarheel sedge	species
Carex aztecica Mack.	Aztec sedge	species
Carex backii Boott	Back's sedge	species
Carex baileyi Britton	Bailey's sedge	species
Carex baltzellii Chapm.	Baltzell's sedge	species
Carex barrattii Torr. ex Schwein.	Barratt's sedge	species
Carex bebbii (L. H. Bailey) Olney ex Fernald	Bebb's sedge	species
Carex bella L.H. Bailey	southwestern showy sedge	species
Carex bicknellii Britton & A.Br.	Bicknell's sedge	species
Carex bicolor Bellardi ex All.	two-color sedge	species
Carex bigelowii Torr. ex Schwein.	Bigelow's sedge	species
Carex biltmoreana Mack.	stiff sedge	species
Carex blanda Dewey	eastern woodland sedge	species
Carex bolanderi Olney	Bolander's sedge	species
Carex boliviensis Van Heurck & Müll. Arg.	Bolivian sedge	species
Carex breweri Boott	Brewer's sedge	species
Carex brizoides L.		species
Carex bromoides Willd.	brome-like sedge	species
Carex brunnescens (Pers.) Poir.	brownish sedge	species
Carex bullata Willd.	button sedge	species
Carex bushii Mack.	Bush's sedge	species
Carex buxbaumii Wahlenb.	Buxbaum's sedge	species
Carex californica L.H. Bailey	California sedge	species
Carex canescens L.	silvery sedge	species
Carex capillaris L.	hair-like sedge	species
Carex capitata Sol.	capitate sedge	species
Carex careyana Torr. ex Dewey	Carey's sedge	species
Carex caroliniana Schwein.	Carolina sedge	species
Carex caryophyllea Latourr.	vernal sedge	species
Carex castanea Wahlenb.	chestnut sedge	species
Carex cephaloidea (Dewey) Dewey ex Boott	thinleaf sedge	species
Carex cephalophora Muhl. ex Willd.	oval-leaf sedge	species
Carex cherokeensis Schwein.	Cherokee sedge	species
Carex chihuahuensis Mack.	Chihuahuan sedge	species
Carex chordorrhiza L.	creeping sedge	species
Carex circinnata C.A.Mey.	coiled sedge	species
Carex collinsii Nutt.	Collins' sedge	species
Carex communis L.H. Bailey	fibrousroot sedge	species
Carex comosa Boott	longhair sedge	species
Carex complanata Torr. & Hook.	hirsute sedge	species
Carex concinna R. Br.	low northern sedge	species
Carex concinnoides Mack.	northwestern sedge	species
Carex conjuncta Boott	soft fox sedge	species
Carex conoidea Willd.	openfield sedge	species
Carex crawei Dewey ex Torr.	Crawe's sedge	species
Carex crawfordii Fernald	Craweford's sedge	species
Carex crebriflora Wiegand	coastal plain sedge	species
Carex crinita Lam.	fringed sedge	species
Carex cristatella Britton & A.Br.	crested sedge	species
Carex crus-corvi Shuttlew. ex Kunze	ravenfoot sedge	species
Carex cryptolepis Mack.	northeastern sedge	species
Carex cumulata (L.H. Bailey) Mack.	clustered sedge	species
Carex cusickii Mack.	Cusick's sedge	species
Carex dasycarpa Muhl.	sandywoods sedge	species
Carex davisii Schwein. & Torr.	Davis' sedge	species
Carex davyi Mack.	Davy's sedge	species
Carex debilis Michx.	white edge sedge	species
Carex decomposita Muhl.	cypressknee sedge	species
Carex deflexa Hornem.	northern sedge	species
Carex densa (L.H. Bailey) L.H. Bailey	dense sedge	species
Carex deweyana Schwein.	Dewey's sedge	species
Carex diandra Schrank	lesser panicled sedge	species
Carex digitalis Willd.	slender woodland sedge	species
Carex donnell-smithii L.H. Bailey	Donell's sedge	species
Carex douglasii Boott	Douglas' sedge	species
Carex ebenea Rydb.	ebony sedge	species
Carex eburnea Boott	bristleleaf sedge	species
Carex egglestonii Mack.	Eggleston's sedge	species
Carex elliottii Schwein. & Torr.	Elliott's sedge	species
Carex elynoides Holm	blackroot sedge	species
Carex emoryi Dewey	Emory's sedge	species
Carex engelmannii L.H. Bailey	Engelmann's sedge	species
Carex exilis Dewey	coastal sedge	species
Carex exsiccata L.H. Bailey	western inflated sedge	species
Carex festucacea Schkuhr ex Willd.	fescue sedge	species
Carex feta L. H. Bailey	greensheath sedge	species
Carex filifolia Nutt.	threadleaf sedge	species
Carex fissa Mack.	hammock sedge	species
Carex flacca Schreb.	heath sedge	species
Carex flaccosperma Dewey	thinfruit sedge	species
Carex flava L.	yellow sedge	species
Carex floridana Schwein.	Florida sedge	species
Carex foenea Willd.	dry-spike sedge	species
Carex folliculata L.	norther long sedge	species
Carex formosa Dewey	handsome sedge	species
Carex fracta Mack.	fragile sheath sedge	species
Carex frankii Kunth	Frank's sedge	species
Carex garberi Fernald	elk sedge	species
Carex geophila Mack.	White Mountain sedge	species
Carex geyeri Boott	Geyer's sedge	species
Carex gigantea Rudge	giant sedge	species
Carex glacialis Mack.	glacial sedge	species
Carex glareosa Schkuhr ex Wahlenb.	lesser salt marsh sedge	species
Carex glaucescens Elliott	southern waxy sedge	species
Carex glaucodea Tuck. ex Olney	blue sedge	species
Carex globosa Boott	roundfruit sedge	species
Carex gmelinii Hook. & Arn.	Gmelin's sedge	species
Carex gracillima Schwein.	graceful sedge	species
Carex granularis Muhl. ex Willd.	limestone meadow sedge	species
Carex gravida L.H. Bailey	heavy sedge	species
Carex grayi J. Carey	Gray's sedge	species
Carex grisea Wahlenb.	inflated narrow-leaf sedge	species
Carex gynandra Schwein.	nodding sedge	species
Carex gynocrates Wormsk.	northern bog sedge	species
Carex gynodynama Olney	Olney's hairy sedge	species
Carex halliana L.H. Bailey	Hall's sedge	species
Carex hallii Olney	deer sedge	species
Carex harfordii Mack.	Harford's sedge	species
Carex hassei L.H. Bailey	salt sedge	species
Carex haydenii Dewey	Hayden's sedge	species
Carex helleri Mack.	Heller's sedge	species
Carex hendersonii L. H. Bailey	Henderson's sedge	species
Carex heteroneura S.Watson	different-nerve sedge	species
Carex hirsutella Mack.	fuzzy sedge	species
Carex hirta L.	hammer sedge	species
Carex hirtifolia Mack.	pubescent sedge	species
Carex hirtissima W. Boott	fuzzy sedge	species
Carex hitchcockiana Dewey	Hitchcock's sedge	species
Carex holostoma Drejer	arctic marsh sedge	species
Carex hoodii Boott	Hood's sedge	species
Carex hookeriana Dewey	Hooker's sedge	species
Carex hormathodes Fernald	marsh straw sedge	species
Carex houghtoniana Torr. ex Dewey	Houghton's sedge	species
Carex hyalina Boott	tissue sedge	species
Carex hyalinolepis Steud	shoreline sedge	species
Carex hystericina Muhl. ex Willd.	bottlebrush sedge	species
Carex idahoa L. H. Bailey	Idaho sedge	species
Carex illota L. H. Bailey	sheep sedge	species
Carex incurviformis Mack.	coastal sand sedge	species
Carex inops L. H. Bailey	long-stolon sedge	species
Carex integra Mack.	smoothbeak sedge	species
Carex interior L. H. Bailey	inland sedge	species
Carex interrupta Boeckeler	greenfruit sedge	species
Carex intumescens Rudge	greater bladder sedge	species
Carex jamesii Schwein.	James' sedge	species
Carex jonesii L.H. Bailey	Jones' sedge	species
Carex joorii L.H. Bailey	cypress swamp sedge	species
Carex lacustris Willd.	hairy sedge? (lake sedge)	species
Carex laeviculmis Meinsh.	smoothstem sedge	species
Carex laxiculmis Schwein.	spreading sedge	species
Carex laxiflora Lam.	broad looseflower sedge	species
Carex leavenworthii Dewey	Leavenworth's sedge	species
Carex lemmonii W. Boott	Lemmon's sedge	species
Carex lenticularis Michx.	lakeshore sedge	species
Carex leporinella Mack.	Sierra hare sedge	species
Carex leptalea Wahlenb.	bristlystalked sedge	species
Carex leptonervia (Fernald) Fernald	nerveless woodland sedge	species
Carex limosa L.	mud sedge	species
Carex livida (Wahlenb.) Willd.	livid sedge	species
Carex loliacea L.	ryegrass sedge	species
Carex lonchocarpa Willd. ex Spreng.	southern long sedge	species
Carex longii Mack.	Long's sedge	species
Carex louisianica L. H. Bailey	Louisiana sedge	species
Carex lucorum Willd.	Blue Ridge sedge	species
Carex lupuliformis Sartwell ex Dewey	false hop sedge	species
Carex lupulina Muhl. ex Willd.	hop sedge	species
Carex lurida Wahlenb.	shallow sedge	species
Carex luzulina Olney	woodrush sedge	species
Carex lyngbyei Hornem.	Lyngbye's sedge	species
Carex macloviana d'Urv.	thickhead sedge	species
Carex macrocephala Willd. ex Spreng.	largehead sedge	species
Carex macrochaeta C. A. Mey.	longawn sedge	species
Carex marina Dewey	sea sedge	species
Carex mariposana L.H. Bailey ex Mack.	Mariposa sedge	species
Carex meadii Dewey	Mead's sedge	species
Carex membranacea Hook.	fragile sedge	species
Carex merritt-fernaldii Mack.	Fernald's sedge	species
Carex mertensii Prescott ex Bong.	Mertens' sedge	species
Carex michauxiana Boeckeler	Michaux's sedge	species
Carex microdonta Torr.	littletooth sedge	species
Carex microglochin Wahlenb.	fewseeded bog sedge	species
Carex micropoda C. A. Mey.		species
Carex microptera Mack.	small wing sedge	species
Carex misera Buckley	wretched sedge	species
Carex mitchelliana M. A. Curtis	Mitchell's sedge	species
Carex molesta Mack.	troublesome sedge	species
Carex muehlenbergii Willd.	Muehlenberg's sedge	species
Carex multicaulis L.H. Bailey	manystem sedge	species
Carex multicostata Mack.	manyrib sedge	species
Carex muricata L.	rough sedge	species
Carex muskingumensis Schwein.	Muskingum sedge	species
Carex nebraskensis Dewey	Nebraska sedge	species
Carex nervina L.H. Bailey	Sierra sedge	species
Carex neurophora Mack.	alpine nerve sedge	species
Carex nigromarginata Schwein.	black edge sedge	species
Carex normalis Mack.	greater straw sedge	species
Carex norvegica Retz.	Norway sedge	species
Carex nudata W. Boott	naked sedge	species
Carex obnupta L. H. Bailey	slough sedge	species
Carex obtusata Lilj.	obtuse sedge	species
Carex occidentalis L. H. Bailey	western sedge	species
Carex oligosperma Michx.	fewseed sedge	species
Carex oreocharis Holm	grassyslope sedge	species
Carex ormostachya Wiegand	necklace spike sedge	species
Carex oxylepis Torr. & Hook.	sharpscale sedge	species
Carex paleacea Schreb. ex Wahlenb.	chaffy sedge	species
Carex pallescens L.	pale sedge	species
Carex panicea L.	grass-like sedge	species
Carex pansa L.H. Bailey	Payson's sedge	species
Carex pauciflora Lightf.	fewflower sedge	species
Carex peckii Howe	Peck's sedge	species
Carex pedunculata Muhl. ex Willd.	longstalk sedge	species
Carex pellita Muhl ex Willd.	wooly sedge	species
Carex pensylvanica Lam.	Pensylvania sedge	species
Carex perglobosa Mack.	globe sedge	species
Carex petricosa Dewey	rockdwelling sedge	species
Carex phaeocephala Piper	dunhead sedge	species
Carex picta Steud.	Boott's sedge	species
Carex pityophila Mack.	loving sedge	species
Carex planostachys Kunze	cedar sedge	species
Carex plantaginea Lam.	plantainleaf sedge	species
Carex platyphylla J. Carey	broadleaf sedge	species
Carex podocarpa R. Br.	shortstalk sedge	species
Carex polystachya Sw. ex Wahlenb.	Caribbean sedge	species
Carex praeceptorium Mack.	early sedge	species
Carex praegracilis W. Boott	clustered field sedge	species
Carex prairea Dewey ex Alph.Wood	prairie sedge	species
Carex prasina Wahlenb.	drooping sedge	species
Carex praticola Rydb.	meadow sedge	species
Carex preslii Steud.	Presl's sedge	species
Carex projecta Mack.	necklace sedge	species
Carex proposita Mack.	Great Smoky Mountain sedge	species
Carex pseudocyperus L.	cypress-like sedge	species
Carex purpurifera Mack.	purple sedge	species
Carex radiata (Wahlenb.) Small	eastern star sedge	species
Carex rariflora (Wahlenb.) Sm.	looseflower alpine sedge	species
Carex raynoldsii Dewey	Raynolds' sedge	species
Carex recta Boott	estuary sedge	species
Carex reniformis (L.H. Bailey) Small	kidneyshape sedge	species
Carex retroflexa Muhl. ex Willd.	reflexed sedge	species
Carex rosea Willd.	rosy sedge	species
Carex rossii Boott	Ross' sedge	species
Carex rostrata Stokes	beaked sedge	species
Carex rufina Drejer	snowbed sedge	species
Carex rupestris All.	curly sedge	species
Carex sartwellii Dewey	Sartwell's sedge	species
Carex saxatilis L.	rock sedge	species
Carex scabrata Schwein.	eastern rough sedge	species
Carex scabriuscula Mack.	Siskiyou sedge	species
Carex schweinitzii Dewey ex Schwein.	Schweinitz's sedge	species
Carex scirpoidea Michx.	northern singlespike sedge	species
Carex scoparia Willd.	broom sedge	species
Carex scopulorum Holm	mountain sedge	species
Carex senta Boott	swamp carex	species
Carex seorsa Howe	weak stellate sedge	species
Carex shortiana Dewey & Torr.	Short's sedge	species
Carex simulata Mack.	analogue sedge	species
Carex socialis Mohlenbr. & Schwegman	low woodland sedge	species
Carex sparganioides Muhl. ex Willd.	bur-reed sedge	species
Carex specifica L.H. Bailey	narrowfruit sedge	species
Carex spectabilis Dewey	showy sedge	species
Carex spicata Huds.	prickly sedge	species
Carex spissa L.H.Bailey ex Hemsl.	San Diego sedge	species
Carex sprengelii Dewey ex Spreng.	Sprengel's sedge	species
Carex squarrosa L.	squarrose sedge	species
Carex sterilis Willd.	dioecious sedge	species
Carex stipata Muhl. ex Willd.	awlfruit sedge	species
Carex straminea Willd. ex Schkuhr	straw sedge	species
Carex striata Michx.	Walter's sedge	species
Carex striatula Michx.	lined sedge	species
Carex stricta Lam.	upright sedge	species
Carex styloflexa Buckley	bent sedge	species
Carex stylosa C. A. Mey.	variegated sedge	species
Carex subbracteata Mack.	smallbract sedge	species
Carex supina Willd. ex Wahlenb.	weak arctic sedge	species
Carex swanii (Fernald) Mack.	Swan's sedge	species
Carex sylvatica Huds.	European woodland sedge	species
Carex tenera Dewey	quill sedge	species
Carex tetanica Schkuhr	rigid sedge	species
Carex torreyi Tuck.	Torrey's sedge	species
Carex tribuloides Wahlenb.	blunt broom sedge	species
Carex tuckermanii Boott	Tuckerman's sedge	species
Carex turgescens Torr.	pine barren sedge	species
Carex typhina Michx.	cattail sedge	species
Carex umbellata Willd.	parasol sedge	species
Carex verrucosa Muhl.	warty sedge	species
Carex vesicaria L.	blister sedge	species
Carex viridula Michx.	little green sedge	species
Carex vulpina L.	true-fox sedge	species
Carex vulpinoidea Michx.	fox sedge	species
Carex willdenowii Willd.	Willdenow's sedge	species
Carex woodii Dewey	Wood's sedge	species
Carex xerantica L.H. Bailey	whitescale sedge	species
Cymophyllus fraseri (Ker Gawl.) Kartesz & Gandhi	Fraser's cymophyllous	species
Kobresia simpliciuscula (Wahlenb.) Mack.	simple bog sedge	species

## Usage rights

### Use license

Open Data Commons Attribution License

## Characters used in the key

1. Species
2. Country
3. U.S. state
4. Canadian province or territory
5. Section within Carex
6. Culm height
7. Blade width
8. Inflorescence type
9. Proximal spike sexuality
10. Terminal spike sexuality
11. Stigma branch number
12. Perigynium length
13. Perigynium width
14. Perigynium cross-section shape
15. Achene length
16. Achene width
17. Achene cross-section shape
18. Style: whether deciduous or persistent
19. Image author
20. Image type

## Software specification

### Name

Carices Interactive Visual Identification Key

### Version

1.1

### Interface language

English

### Platform

Silverlight runtime

### Web location

http://www.herbarium2.lsu.edu/aba/

## Software technical features

**Main XAML page**

<UserControl x:Class="A5.MainPage"

    xmlns="http://schemas.microsoft.com/winfx/2006/xaml/presentation"

    xmlns:x="http://schemas.microsoft.com/winfx/2006/xaml"

    xmlns:d="http://schemas.microsoft.com/expression/blend/2008"

    xmlns:mc="http://schemas.openxmlformats.org/markup-compatibility/2006"

    xmlns:local="clr-namespace:System.Windows.Pivot;assembly=System.Windows.Pivot"

    mc:Ignorable="d" d:DesignHeight="300" d:DesignWidth="400" Loaded="UserControl_Loaded">

  <Grid x:Name="LayoutRoot" Background="Black">

  <local:PivotViewer x:Name="Pivot"/>

  </Grid>

</UserControl>

**XAML.CS or Code behind**

using System;

using System.Collections.Generic;

using System.Linq;

using System.Net;

using System.Windows;

using System.Windows.Controls;

using System.Windows.Documents;

using System.Windows.Input;

using System.Windows.Media;

using System.Windows.Media.Animation;

using System.Windows.Shapes;

using System.Windows.Pivot;

namespace A10

{

  public partial class MainPage: UserControl

  {

    public MainPage()

    {

      InitializeComponent();

      Pivot.LoadCollection("http://www.herbarium2.lsu.edu/aba/A10.cxml", string.Empty);

    }

    private void UserControl_Loaded(object sender, RoutedEventArgs e)

    {

    }

  }

}

## Additional information

Later examples of visual keys deal with the clustering problem differently. Both Silverlight and HTML 5 based grass genera of Louisiana keys use existing herbarium specimen images to normalize, one herbarium specimen per taxon. Leveraging recent physical and vetted sources. This normalization character is select-able as 'one-to-one comparisons' at the bottom of character information panel http://www.herbarium2.lsu.edu/grass2/. Secondly, Kingdom Plantae in HTML 5 is normalized by image number only, without a selectable character state, across divisions http://www.herbarium2.lsu.edu/aca/. *Magnoliophyta* is taken at a log value due to its disparate taxa value when compared to the other divisions.

## Figures and Tables

**Figure 4.** An Interactive Visual Identification Key to Carices of North America beta version.

## Supplementary files

### Tertiary file structure for Carices CXML file

**Authors:** Jones, T. M. **Data type:** occurences, morphological, **File:** A10.cxml

### Secondary Carex morphology data; cleaned and truncated for building CXML

**Authors:** Jones, T. M. **Data type:** occurrences, morphological, images This file is an example of a build file for the creation of the CXML file. **File:** 957am fixed scirpoidea space issue.xlsx

### Website data from Utah State University

**Authors:** Google Analytics **Data type:** PDF Data sheet for visitiation to CIIK by country **File:** Analytics utc.usu.edu_keys_Carex_Carex.html Location 20060531-20130630.pdf

### Website data from Louisiana State University

**Authors:** Google Analytics **Data type:** PDF Data sheet for visitiation to CIIK by country **File:** Analytics Carex key LSU Location 20060531-20130630.pdf

### Primary Carex morphology data from Lucid 3.4

**Authors:** Jones, T. M. **Data type:** XLSX Export from CIIK 2013 in CSV format **File:** Carex-all-CSV.xlsx

### CIVIK usage 2011 - 2013

**Authors:** Google Analytics **Data type:** PDF This includes all visual keys developed. Here CIVIK is represented by both /aba/ and /aaa/ and iteratives. **File:** Analytics www.herbarium2.lsu.edu_aaa_A5TestPage.html Pages 20100531-20130630.pdf

### Visual keys usage with Google Analytics

**Authors:** Google **Data type:** analytics Compilation of all visual keys using Google Analytics **File:** Analytics www.herbarium2.lsu.edu-aaa-A5TestPage.html Language 20100809-20130908.pdf
