# Supplementary material for: A visual identification key utilizing both gestalt and analytic approaches to identification of Carices present in North America (Plantae, Cyperaceae)
Source: Biodivers Data J. 2013 Sep 16;(1):e984. doi: 10.3897/BDJ.1.e984 (PMC3964697; doi:10.3897/BDJ.1.e984)
Supplement: Supplementary file 3 — Authors: Google Analytics Data type: PDF Data sheet for visitiation to CIIK by country File: Analytics utc.usu.edu_keys_Carex_Carex.html Location 20060531-20130630.pdf [file biodiversity_data_journal-1-e984-s003.pdf]

Google Analytics

Go to this report

http://utc.usu.edu/keys/Carex/Carex.html - http://utc.usu.edu/…
utc.usu.edu/keys/Carex/Carex.html [DEFAULT]

Location

May 31, 2006 - Jun 30, 2013

% of visits: 100.00%

Map Overlay

Site Usage

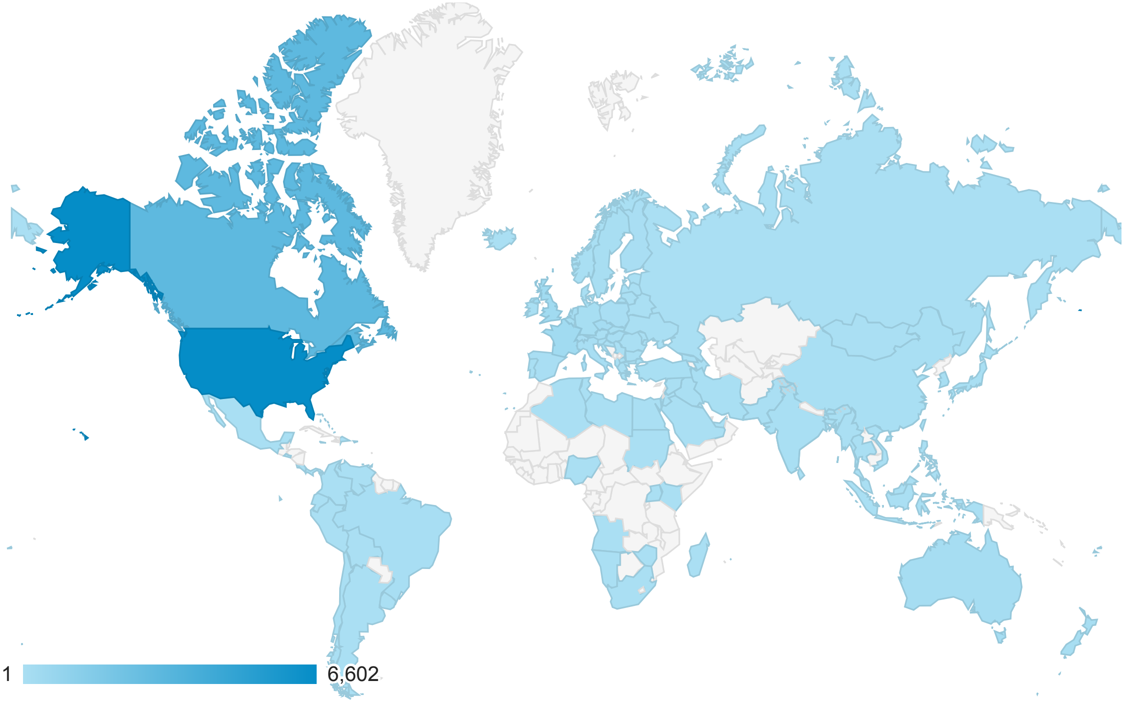

Country / Territory	Visits	Pages / Visit	Avg. Visit Duration	% New Visits	Bounce Rate
	**12,524** % of Total: 100.00% (12,524)	**1.25** Site Avg: 1.25 (0.00%)	**00:01:02** Site Avg: 00:01:02 (0.00%)	**75.77%** Site Avg: 75.77% (0.00%)	**82.92%** Site Avg: 82.92% (0.00%)
1. United States	**6,602**	1.26	00:01:11	68.62%	82.35%
2. Canada	**3,036**	1.23	00:00:55	85.90%	84.62%
3. United Kingdom	**356**	1.21	00:00:31	82.02%	85.11%
4. France	**345**	1.23	00:00:46	94.49%	83.48%
5. Czech Republic	**215**	1.20	00:00:31	77.67%	84.65%
6. Australia	**160**	1.24	00:00:53	80.00%	83.12%
7. Netherlands	**144**	1.27	00:00:55	82.64%	81.25%
8. Belgium	**110**	1.25	00:00:53	82.73%	79.09%
9. Germany	**103**	1.16	00:00:34	87.38%	85.44%
10. Sweden	**96**	1.29	00:00:47	71.88%	78.12%
11. New Zealand	**86**	1.43	00:01:16	53.49%	69.77%
12. South Korea	**80**	1.44	00:01:54	47.50%	78.75%
13. Hungary	**72**	1.31	00:01:02	88.89%	80.56%
14. Brazil	**64**	1.19	00:00:47	81.25%	82.81%
15. Italy	**63**	1.33	00:00:32	93.65%	79.37%
16. Spain	**58**	1.16	00:00:33	77.59%	91.38%
17. Finland	**51**	1.18	00:01:03	64.71%	82.35%

18.	Switzerland	**47**	1.23	00:00:59	85.11%	80.85%
19.	Poland	**47**	1.19	00:00:14	80.85%	80.85%
20.	India	**41**	1.41	00:01:52	85.37%	78.05%
21.	Portugal	**38**	1.47	00:01:10	76.32%	73.68%
22.	Mexico	**36**	1.33	00:00:45	83.33%	80.56%
23.	China	**31**	1.13	00:00:21	77.42%	93.55%
24.	Turkey	**30**	1.17	00:00:12	93.33%	90.00%
25.	Taiwan	**30**	1.23	00:00:35	50.00%	80.00%
26.	Slovakia	**27**	1.37	00:00:20	92.59%	70.37%
27.	Japan	**25**	1.20	00:00:57	76.00%	84.00%
28.	Denmark	**23**	1.22	00:00:34	86.96%	86.96%
29.	Norway	**23**	1.17	00:00:14	78.26%	91.30%
30.	Ireland	**22**	1.23	00:02:16	95.45%	81.82%
31.	Iran	**22**	1.50	00:01:01	72.73%	77.27%
32.	Latvia	**20**	1.25	00:05:18	45.00%	80.00%
33.	Slovenia	**18**	1.22	00:00:23	88.89%	83.33%
34.	Romania	**17**	1.41	00:02:07	94.12%	76.47%
35.	Vietnam	**17**	1.29	00:02:51	82.35%	70.59%
36.	Greece	**16**	1.31	00:01:11	93.75%	75.00%
37.	Indonesia	**15**	1.20	00:00:43	93.33%	80.00%
38.	Croatia	**14**	1.21	00:00:45	85.71%	78.57%
39.	Serbia	**14**	1.07	00:00:04	92.86%	92.86%
40.	Austria	**13**	1.15	00:00:05	84.62%	84.62%
41.	Colombia	**13**	1.23	00:02:49	92.31%	76.92%
42.	Philippines	**13**	1.08	00:00:12	84.62%	92.31%
43.	Argentina	**12**	1.50	00:02:09	91.67%	75.00%
44.	Estonia	**12**	1.08	00:00:02	75.00%	91.67%
45.	Russia	**12**	1.08	00:00:18	91.67%	91.67%
46.	Thailand	**12**	1.17	00:00:09	91.67%	83.33%
47.	Malaysia	**11**	1.27	00:00:27	100.00%	72.73%
48.	Kenya	**10**	1.00	00:00:00	10.00%	100.00%
49.	South Africa	**10**	1.20	00:00:06	100.00%	80.00%
50.	Costa Rica	**9**	1.11	00:02:41	88.89%	88.89%
51.	Pakistan	**9**	1.00	00:00:00	88.89%	100.00%
52.	Ecuador	**8**	1.38	00:00:11	50.00%	62.50%
53.	Nigeria	**8**	1.12	00:00:07	100.00%	87.50%
54.	Chile	**7**	1.14	00:00:19	100.00%	85.71%
55.	Egypt	**7**	1.29	00:00:11	85.71%	71.43%
56.	Israel	**7**	1.00	00:00:00	85.71%	100.00%
57.	Peru	**7**	1.43	00:00:15	100.00%	71.43%

58. Venezuela	**7**	1.00	00:00:00	57.14%	100.00%
59. Serbia	**6**	1.00	00:00:00	66.67%	100.00%
60. Mauritius	**6**	1.00	00:00:00	66.67%	100.00%
61. Ukraine	**6**	1.50	00:00:47	100.00%	66.67%
62. Uruguay	**6**	1.17	00:02:42	100.00%	83.33%
63. (not set)	**6**	1.33	00:00:04	83.33%	83.33%
64. Bulgaria	**5**	1.40	00:00:19	100.00%	60.00%
65. Iceland	**5**	1.20	00:00:03	100.00%	80.00%
66. Lithuania	**5**	1.00	00:00:00	100.00%	100.00%
67. Saudi Arabia	**5**	1.00	00:00:00	100.00%	100.00%
68. Sudan	**5**	1.00	00:00:00	40.00%	100.00%
69. Algeria	**4**	1.50	00:02:22	100.00%	50.00%
70. Singapore	**4**	1.00	00:00:00	100.00%	100.00%
71. Belarus	**3**	2.33	00:02:36	33.33%	33.33%
72. Jordan	**3**	1.67	00:01:07	100.00%	66.67%
73. Sri Lanka	**3**	1.00	00:00:00	66.67%	100.00%
74. Puerto Rico	**3**	1.00	00:00:00	100.00%	100.00%
75. Syria	**3**	1.00	00:00:00	100.00%	100.00%
76. United Arab Emirates	**2**	1.00	00:00:00	100.00%	100.00%
77. Azerbaijan	**2**	1.50	00:02:08	100.00%	50.00%
78. Hong Kong	**2**	1.00	00:00:00	100.00%	100.00%
79. Jamaica	**2**	1.00	00:00:00	100.00%	100.00%
80. Madagascar	**2**	1.00	00:00:00	100.00%	100.00%
81. Macedonia [FYROM]	**2**	1.00	00:00:00	100.00%	100.00%
82. Malta	**2**	1.00	00:00:00	100.00%	100.00%
83. Namibia	**2**	1.50	00:05:45	100.00%	50.00%
84. Zimbabwe	**2**	1.50	00:01:19	100.00%	50.00%
85. Anguilla	**1**	1.00	00:00:00	100.00%	100.00%
86. Albania	**1**	1.00	00:00:00	100.00%	100.00%
87. Angola	**1**	1.00	00:00:00	100.00%	100.00%
88. Barbados	**1**	1.00	00:00:00	100.00%	100.00%
89. Bangladesh	**1**	1.00	00:00:00	100.00%	100.00%
90. Bermuda	**1**	2.00	00:01:22	100.00%	0.00%
91. Bolivia	**1**	1.00	00:00:00	100.00%	100.00%
92. Bahamas	**1**	1.00	00:00:00	100.00%	100.00%
93. Bhutan	**1**	1.00	00:00:00	100.00%	100.00%
94. Dominican Republic	**1**	1.00	00:00:00	100.00%	100.00%
95. Fiji	**1**	1.00	00:00:00	100.00%	100.00%
96. Georgia	**1**	2.00	00:01:44	100.00%	0.00%
97. Guernsey	**1**	1.00	00:00:00	100.00%	100.00%
98. Iraq	**1**	1.00	00:00:00	100.00%	100.00%

98.	Iraq	**1**	1.00	00:00:00	100.00%	100.00%
99.	Jersey	**1**	1.00	00:00:00	100.00%	100.00%
100.	Kuwait	**1**	1.00	00:00:00	100.00%	100.00%
101.	Luxembourg	**1**	1.00	00:00:00	0.00%	100.00%
102.	Libya	**1**	1.00	00:00:00	100.00%	100.00%
103.	Moldova	**1**	1.00	00:00:00	0.00%	100.00%
104.	Myanmar [Burma]	**1**	1.00	00:00:00	100.00%	100.00%
105.	Mongolia	**1**	1.00	00:00:00	100.00%	100.00%
106.	Panama	**1**	1.00	00:00:00	100.00%	100.00%
107.	El Salvador	**1**	2.00	00:00:10	100.00%	0.00%
108.	Tunisia	**1**	2.00	00:00:45	100.00%	0.00%
109.	Trinidad and Tobago	**1**	1.00	00:00:00	100.00%	100.00%
110.	Uganda	**1**	2.00	00:17:15	100.00%	0.00%
111.	Saint Vincent and the Grenadines	**1**	1.00	00:00:00	100.00%	100.00%

Rows 1 - 111 of 111

© 2013 Google
