# Supplementary material for: A visual identification key utilizing both gestalt and analytic approaches to identification of Carices present in North America (Plantae, Cyperaceae)
Source: Biodivers Data J. 2013 Sep 16;(1):e984. doi: 10.3897/BDJ.1.e984 (PMC3964697; doi:10.3897/BDJ.1.e984)
Supplement: Supplementary file 4 — Authors: Google Analytics Data type: PDF Data sheet for visitiation to CIIK by country File: Analytics Carex key LSU Location 20060531-20130630.pdf [file biodiversity_data_journal-1-e984-s004.pdf]

Google Analytics

Go to this report

http://www.herbarium.lsu.edu - http://www.herbarium.lsu.edu
Carex key LSU [DEFAULT]

Location

May 31, 2006 - Jun 30, 2013

% of visits: 100.00%

Map Overlay

Site Usage

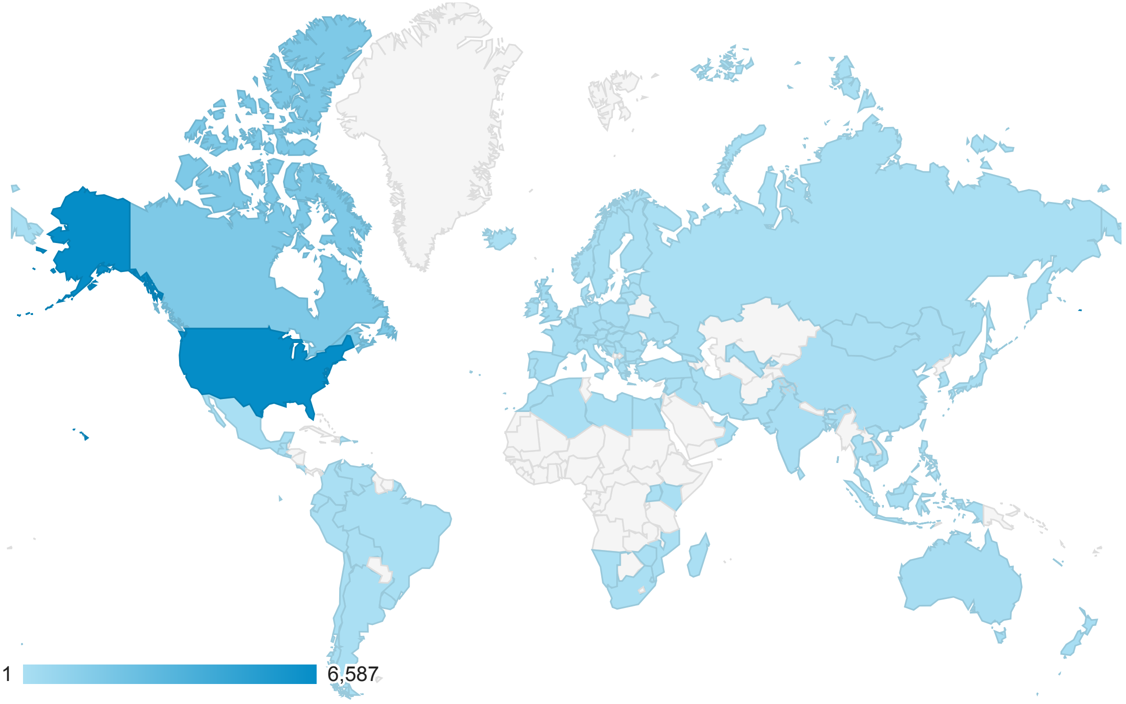

Country / Territory	Visits	Pages / Visit	Avg. Visit Duration	% New Visits	Bounce Rate
	**10,359** % of Total: **100.00%** (10,359)	**1.46** Site Avg: **1.46** (0.00%)	**00:01:52** Site Avg: **00:01:52** (0.00%)	**59.17%** Site Avg: **59.22%** (-0.10%)	**73.79%** Site Avg: **73.79%** (0.00%)
1. United States	**6,587**	1.51	00:02:18	56.90%	71.63%
2. Canada	**1,768**	1.33	00:01:08	48.19%	79.64%
3. United Kingdom	**306**	1.20	00:00:50	59.80%	84.97%
4. France	**169**	1.40	00:01:40	84.62%	74.56%
5. Australia	**134**	1.28	00:00:47	71.64%	79.85%
6. South Korea	**94**	1.50	00:00:57	67.02%	70.21%
7. Netherlands	**91**	1.43	00:00:40	72.53%	70.33%
8. Spain	**75**	1.45	00:01:03	68.00%	80.00%
9. Germany	**65**	1.37	00:01:31	75.38%	78.46%
10. New Zealand	**65**	1.38	00:00:59	72.31%	72.31%
11. India	**60**	1.48	00:01:06	70.00%	73.33%
12. Poland	**59**	1.24	00:00:53	83.05%	79.66%
13. Czech Republic	**57**	1.46	00:01:55	80.70%	78.95%
14. Belgium	**56**	1.54	00:01:25	91.07%	69.64%
15. China	**49**	1.24	00:00:21	89.80%	87.76%
16. Brazil	**39**	1.33	00:00:45	74.36%	71.79%
17. Italy	**39**	1.49	00:01:01	92.31%	69.23%

18.	Switzerland	**35**	1.57	00:00:58	74.29%	71.43%
19.	Hungary	**32**	1.19	00:00:08	100.00%	84.38%
20.	Russia	**31**	1.68	00:01:20	54.84%	61.29%
21.	Serbia	**28**	1.50	00:00:43	50.00%	75.00%
22.	Mexico	**27**	1.56	00:01:11	74.07%	66.67%
23.	(not set)	**24**	1.62	00:02:38	70.83%	66.67%
24.	Sweden	**22**	1.82	00:00:48	72.73%	81.82%
25.	Ireland	**19**	1.21	00:00:38	89.47%	84.21%
26.	Portugal	**18**	1.56	00:02:21	94.44%	44.44%
27.	Taiwan	**18**	1.44	00:01:09	72.22%	66.67%
28.	Iran	**16**	1.38	00:01:12	93.75%	68.75%
29.	Peru	**16**	1.50	00:01:39	81.25%	56.25%
30.	Japan	**15**	1.53	00:00:51	86.67%	66.67%
31.	Norway	**15**	1.13	00:00:15	93.33%	86.67%
32.	Philippines	**15**	1.40	00:01:26	100.00%	80.00%
33.	Colombia	**14**	1.43	00:03:49	78.57%	71.43%
34.	Finland	**13**	1.38	00:00:23	92.31%	76.92%
35.	Greece	**13**	1.23	00:00:54	69.23%	84.62%
36.	Malaysia	**13**	1.23	00:00:12	92.31%	76.92%
37.	Turkey	**13**	1.54	00:00:25	92.31%	53.85%
38.	Vietnam	**13**	1.62	00:01:23	92.31%	69.23%
39.	Thailand	**12**	1.17	00:00:05	83.33%	83.33%
40.	Argentina	**11**	1.45	00:00:49	81.82%	63.64%
41.	Denmark	**11**	1.36	00:02:23	90.91%	81.82%
42.	Indonesia	**11**	1.45	00:01:04	90.91%	72.73%
43.	Austria	**10**	1.30	00:01:19	90.00%	70.00%
44.	Romania	**10**	1.30	00:01:34	90.00%	80.00%
45.	Slovenia	**10**	1.70	00:02:16	80.00%	70.00%
46.	Slovakia	**10**	2.10	00:00:27	90.00%	60.00%
47.	South Africa	**10**	1.30	00:00:07	100.00%	80.00%
48.	Estonia	**6**	1.17	00:00:02	100.00%	83.33%
49.	Croatia	**6**	4.00	00:06:07	100.00%	16.67%
50.	Lithuania	**6**	2.17	00:04:27	100.00%	33.33%
51.	Latvia	**6**	1.50	00:01:18	33.33%	66.67%
52.	Singapore	**6**	1.00	00:00:00	83.33%	100.00%
53.	Chile	**5**	1.20	00:01:29	100.00%	80.00%
54.	Costa Rica	**5**	1.40	00:00:14	80.00%	60.00%
55.	Pakistan	**5**	1.20	00:00:11	100.00%	80.00%
56.	Ukraine	**5**	1.00	00:00:00	100.00%	100.00%
57.	Uganda	**5**	1.40	00:01:45	60.00%	60.00%

58.	Venezuela	5	2.00	00:00:09	60.00%	60.00%
59.	United Arab Emirates	4	1.25	00:00:46	75.00%	75.00%
60.	Bosnia and Herzegovina	4	1.00	00:00:00	100.00%	100.00%
61.	Algeria	4	1.50	00:01:41	100.00%	50.00%
62.	Ecuador	4	1.00	00:00:00	100.00%	100.00%
63.	Mongolia	4	1.25	00:00:11	75.00%	75.00%
64.	Puerto Rico	4	1.00	00:00:00	100.00%	100.00%
65.	Israel	3	1.00	00:00:00	66.67%	100.00%
66.	Sri Lanka	3	1.00	00:00:00	66.67%	100.00%
67.	Morocco	3	2.00	00:01:43	66.67%	33.33%
68.	Malta	3	1.33	00:00:50	100.00%	66.67%
69.	Uruguay	3	1.33	00:00:30	33.33%	66.67%
70.	Albania	2	1.50	00:01:08	100.00%	50.00%
71.	Bangladesh	2	1.00	00:00:00	100.00%	100.00%
72.	Bulgaria	2	1.00	00:00:00	100.00%	100.00%
73.	Bolivia	2	4.50	00:11:08	100.00%	0.00%
74.	Egypt	2	1.00	00:00:00	100.00%	100.00%
75.	French Guiana	2	1.00	00:00:00	100.00%	100.00%
76.	Iraq	2	1.00	00:00:00	100.00%	100.00%
77.	Iceland	2	1.00	00:00:00	100.00%	100.00%
78.	Madagascar	2	1.50	00:00:07	100.00%	50.00%
79.	Bahrain	1	2.00	00:00:14	100.00%	0.00%
80.	Bermuda	1	1.00	00:00:00	100.00%	100.00%
81.	Dominican Republic	1	2.00	00:07:36	100.00%	0.00%
82.	Georgia	1	1.00	00:00:00	100.00%	100.00%
83.	Guatemala	1	3.00	00:06:47	100.00%	0.00%
84.	Guam	1	1.00	00:00:00	100.00%	100.00%
85.	Jersey	1	2.00	00:01:41	100.00%	0.00%
86.	Kenya	1	1.00	00:00:00	100.00%	100.00%
87.	Cambodia	1	1.00	00:00:00	100.00%	100.00%
88.	Saint Kitts and Nevis	1	1.00	00:00:00	100.00%	100.00%
89.	Lebanon	1	1.00	00:00:00	100.00%	100.00%
90.	Luxembourg	1	1.00	00:00:00	100.00%	100.00%
91.	Libya	1	2.00	00:01:52	100.00%	0.00%
92.	Macedonia [FYROM]	1	2.00	00:00:14	100.00%	0.00%
93.	Mozambique	1	1.00	00:00:00	100.00%	100.00%
94.	Namibia	1	1.00	00:00:00	100.00%	100.00%
95.	New Caledonia	1	1.00	00:00:00	100.00%	100.00%
96.	Oman	1	1.00	00:00:00	0.00%	100.00%
97.	El Salvador	1	3.00	00:00:50	100.00%	0.00%
98.	Syria	1	1.00	00:00:00	100.00%	100.00%

98.	Syria	**1**	1.00	00:00:00	100.00%	100.00%
99.	Swaziland	**1**	1.00	00:00:00	100.00%	100.00%
100.	Trinidad and Tobago	**1**	1.00	00:00:00	100.00%	100.00%
101.	Uzbekistan	**1**	1.00	00:00:00	100.00%	100.00%
102.	Zimbabwe	**1**	1.00	00:00:00	100.00%	100.00%

Rows 1 - 102 of 102

© 2013 Google
