# Supplementary material for: A visual identification key utilizing both gestalt and analytic approaches to identification of Carices present in North America (Plantae, Cyperaceae)
Source: Biodivers Data J. 2013 Sep 16;(1):e984. doi: 10.3897/BDJ.1.e984 (PMC3964697; doi:10.3897/BDJ.1.e984)
Supplement: Supplementary file 6 — Authors: Google Analytics Data type: PDF This includes all visual keys developed. Here CIVIK is represented by both /aba/ and /aaa/ and iteratives. File: Analytics www.herbarium2.lsu.edu_aaa_A5TestPage.html Pages 20100531-20130630.pdf [file biodiversity_data_journal-1-e984-s006.pdf]

Google Analytics

Go to this report

http://www.herbarium2.lsu.edu/aaa/A5TestPage.html - http://...
www.herbarium2.lsu.edu/aaa/A5TestPage.html [D...

Pages

May 31, 2010 - Jun 30, 2013

Pages are grouped by Page

% of pageviews: 100.00%

Explorer

Site Usage

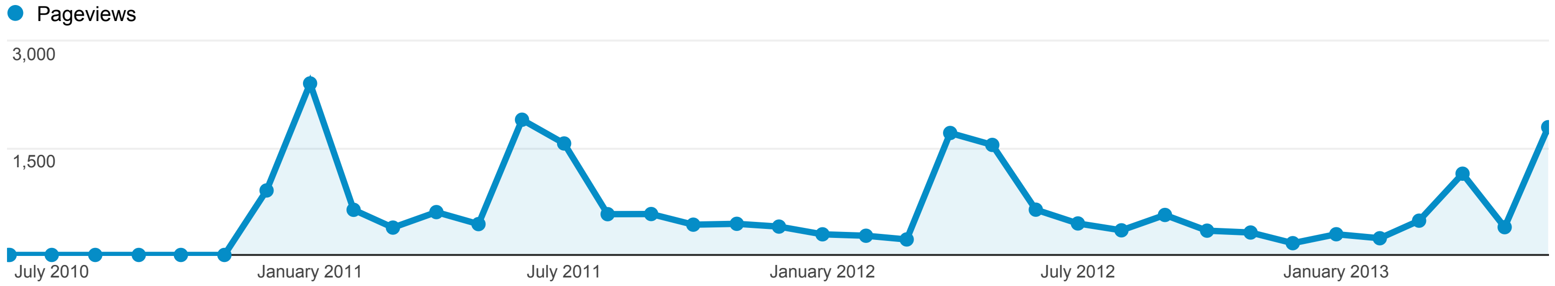

	Page	Pageviews	Unique Pageviews	Avg. Time on Page	Entrances	Bounce Rate	% Exit	Page Value
		**22,351** % of Total: 100.00% (22,351)	**14,040** % of Total: 100.00% (14,040)	**00:03:03** Site Avg: 00:03:03 (0.00%)	**13,279** % of Total: 100.00% (13,279)	**65.74%** Site Avg: 65.74% (0.00%)	**59.41%** Site Avg: 59.41% (0.00%)	**$0.00** % of Total: 0.00% ($0.00)
1.	/grass2/	**7,101**	5,335	00:04:07	5,145	80.86%	72.98%	$0.00
2.	/keys/aca/	**5,118**	1,798	00:01:13	1,780	0.67%	34.66%	$0.00
3.	/aba/	**3,282**	2,306	00:04:49	2,155	72.71%	65.08%	$0.00
4.	/aaa/index.html	**2,866**	2,067	00:03:16	2,027	72.13%	68.39%	$0.00
5.	/aba/index.html	**2,017**	1,315	00:04:23	1,206	68.91%	60.24%	$0.00
6.	/aaa/A5TestPage.html	**481**	379	00:05:41	373	85.52%	75.47%	$0.00
7.	/aca/	**423**	182	00:03:21	141	53.19%	30.73%	$0.00
8.	/bbb/index.html	**317**	243	00:04:03	149	65.77%	58.68%	$0.00
9.	/keys/paspalum/	**209**	170	00:06:03	145	80.00%	71.77%	$0.00
10.	/grass2/index2.html	**104**	53	00:06:03	30	50.00%	41.35%	$0.00
11.	/aca/index2.html	**93**	20	00:02:48	14	35.71%	12.90%	$0.00
12.	/bbb/	**79**	57	00:05:48	47	70.21%	54.43%	$0.00
13.	/aaa/	**49**	26	00:04:39	12	41.67%	36.73%	$0.00
14.	/keys/visual-keys/	**43**	7	00:01:25	6	0.00%	16.28%	$0.00
15.	/keys/aca/index.html	**32**	4	00:02:23	2	0.00%	9.38%	$0.00
16.	/grass/	**29**	16	00:01:58	6	33.33%	27.59%	$0.00
17.	/virtual_classroom/1208lab.html	**22**	20	00:03:42	15	80.00%	86.36%	$0.00
18.	/grass2/grass3.html	**19**	4	00:03:10	0	0.00%	5.26%	$0.00
19.	/grass/index.html	**17**	8	00:02:50	3	66.67%	35.29%	$0.00
20.	/keys/chive/carex-texensis.html	**6**	6	00:00:00	6	100.00%	100.00%	$0.00

21.	/keys/NEW/	**6**	1	00:04:37	1	0.00%	0.00%	$0.00
22.	/translate_c?depth=1&hl=nl&rurl=translate.google.nl&sl=la&tl=nl&twu=1&u=http://www.herbarium.lsu.edu/keys/aca/&usg=ALkJrhg7R2UlShujXrf0YFKZOzMctMnStw	**6**	1	00:00:12	0	0.00%	16.67%	$0.00
23.	/keys/virtual_classroom/1208lab.html	**5**	5	00:00:00	5	100.00%	100.00%	$0.00
24.	/grass2/index.html	**4**	3	00:04:51	0	0.00%	25.00%	$0.00
25.	/grass/grass.html	**3**	1	00:01:46	0	0.00%	0.00%	$0.00
26.	/keys/eee/	**3**	1	00:00:29	1	0.00%	33.33%	$0.00
27.	/translate_c?depth=1&ei=NFf9UI_dDYWr4AS8s4CABg&hl=hr&prev=/search?q=grasses+id&start=130&hl=hr&client=firefox-a&sa=N&tbo=d&rls=org.mozilla:hr:official&biw=1016&bih=612&rurl=translate.google.hr&sl=en&u=http://www.herbarium2.lsu.edu/grass2/&usg=ALkJrhh-OviVxeNETvTjyZrxgRqGoRleAQ	**3**	1	00:00:14	1	0.00%	33.33%	$0.00
28.	/keys/aca/index2.html	**2**	2	00:00:17	1	0.00%	50.00%	$0.00
29.	/translate_c?depth=1&ei=K8JiUeq_LanV4QSY2oGYBw&hl=pl&prev=/search?q=key+of+the+identification&hl=pl&biw=1280&bih=642&rurl=translate.google.pl&sl=en&u=http://www.herbarium2.lsu.edu/grass2/&usg=ALkJrhgHteXz9SHdDjFLz1TNWaIOb0b32w	**2**	1	00:00:12	1	0.00%	50.00%	$0.00
30.	/translate_c?depth=1&ei=V4obUbuWFoTdsgbQ-YHwBQ&hl=nl&prev=/search?q=eys+plantaes&hl=nl&client=safari&tbo=d&biw=1024&bih=672&rurl=translate.google.nl&sl=en&twu=1&u=http://www.herbarium.lsu.edu/keys/aca/&usg=ALkJrhgBm7hYDfwnPCcb2ErH7iBXSWUTvA	**2**	1	00:00:05	1	0.00%	0.00%	$0.00
31.	/translate_c?depth=1&hl=pl&prev=/search?q=animals+key+of+the+identification&hl=pl&biw=1280&bih=642&rurl=translate.google.pl&sl=en&u=http://www.herbarium2.lsu.edu/grass2/&usg=ALkJrhi_kre0B1YcMKm0onn8NRnxV7AkBA	**2**	1	00:00:15	1	0.00%	50.00%	$0.00
32.	/aca/index.html	**1**	1	00:00:00	1	100.00%	100.00%	$0.00
33.	/cache.aspx?q=guide+to+North+American+sedges&d=4889271565946417&mkt=en-US&setlang=en-US&w=vzqxbVIWFOuvYnQg_Lt-ozHal09cOP9H	**1**	1	00:00:00	1	100.00%	100.00%	$0.00
34.	/keys/	**1**	1	00:00:09	0	0.00%	0.00%	$0.00
35.	/search?q=cache:8Qz8ooqZrXkJ:www.herbarium2.lsu.edu/aaa/index.html+interactive+visual+key&cd=7&hl=en&ct=clnk&gl=us	**1**	1	00:00:00	1	100.00%	100.00%	$0.00
36.	/search?q=cache:http://www.herbarium2.lsu.edu/aba/index.html	**1**	1	00:00:00	1	100.00%	100.00%	$0.00
37.	/translate_c?hl=zh-TW&prev=/search?q=植物分類檢索表&hl=zh-TW&biw=1366&bih=587&prmd=ivns&rurl=translate.google.com.tw&sl=zh-CN&twu=1&u=http://www.herbarium2.lsu.edu/grass2/&usg=ALkJrhgoPgi6ml5ZCrgJ6aYZDNwnmsRpEQ	**1**	1	00:00:00	1	100.00%	100.00%	$0.00

Rows 1 - 37 of 37

© 2013 Google
