# Supplementary material for: A visual identification key utilizing both gestalt and analytic approaches to identification of Carices present in North America (Plantae, Cyperaceae)
Source: Biodivers Data J. 2013 Sep 16;(1):e984. doi: 10.3897/BDJ.1.e984 (PMC3964697; doi:10.3897/BDJ.1.e984)
Supplement: Supplementary file 7 — Authors: Google Data type: analytics Compilation of all visual keys using Google Analytics File: Analytics www.herbarium2.lsu.edu-aaa-A5TestPage.html Language 20100809-20130908.pdf [file biodiversity_data_journal-1-e984-s007.pdf]

Google Analytics

Go to this report

http://www.herbarium2.lsu.edu/aaa/A5TestPage.html - http://...
www.herbarium2.lsu.edu/aaa/A5TestPage.html

Aug 9, 2010 - Sep 8, 2013

Language

% of visits: 100.00%

Explorer

Site Usage

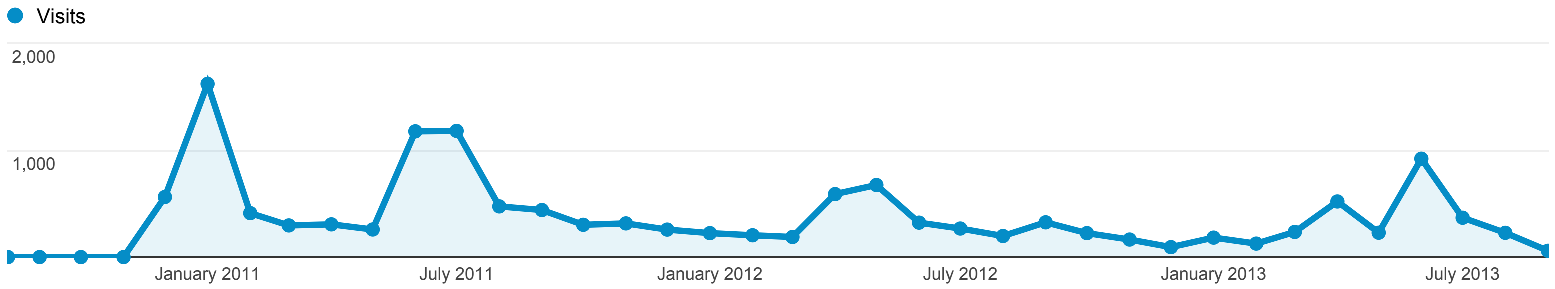

	Language	Visits	Pages / Visit	Avg. Visit Duration	% New Visits	Bounce Rate
		**13,933** % of Total: 100.00% (13,933)	**1.68** Site Avg: 1.68 (0.00%)	**00:02:06** Site Avg: 00:02:06 (0.00%)	**64.67%** Site Avg: 64.65% (0.03%)	**66.08%** Site Avg: 66.08% (0.00%)
1.	en-us	**11,548**	1.74	00:02:21	60.33%	64.07%
2.	en-gb	**281**	1.35	00:00:59	76.51%	76.16%
3.	zh-cn	**209**	1.56	00:00:47	83.73%	72.73%
4.	fr	**207**	1.39	00:00:55	89.86%	74.88%
5.	en	**200**	1.52	00:00:47	65.00%	69.00%
6.	es	**190**	1.36	00:01:24	86.84%	75.26%
7.	ru	**147**	1.14	00:00:25	90.48%	88.44%
8.	es-es	**113**	1.61	00:00:47	81.42%	69.03%
9.	nl	**113**	1.24	00:00:31	88.50%	81.42%
10.	pt-br	**107**	1.36	00:01:38	87.85%	77.57%
11.	de	**98**	1.17	00:00:47	91.84%	86.73%
12.	pl	**72**	1.25	00:00:27	90.28%	79.17%
13.	tr	**66**	1.41	00:00:51	92.42%	80.30%
14.	ko	**56**	1.25	00:00:59	83.93%	80.36%
15.	de-de	**48**	1.25	00:01:05	95.83%	83.33%
16.	ru-ru	**42**	1.29	00:01:00	97.62%	80.95%
17.	zh-tw	**37**	1.24	00:00:06	86.49%	78.38%
18.	ja	**33**	1.42	00:00:53	87.88%	78.79%
19.	it	**32**	1.66	00:01:22	96.88%	56.25%
20.	fr-fr	**21**	1.57	00:00:36	66.67%	57.14%
21.	cs	**19**	1.26	00:02:41	100.00%	78.95%
22.	it-it	**19**	1.21	00:00:00	100.00%	78.95%

23.	es-419	**18**	1.50	00:00:18	77.78%	77.78%
24.	sv-se	**18**	1.33	00:00:17	94.44%	77.78%
25.	da	**17**	1.82	00:01:18	94.12%	76.47%
26.	es-mx	**15**	1.47	00:02:39	100.00%	73.33%
27.	tr-tr	**15**	1.33	00:00:26	100.00%	86.67%
28.	nl-nl	**14**	2.00	00:00:24	92.86%	64.29%
29.	sv	**12**	1.25	00:00:16	91.67%	75.00%
30.	ca	**10**	1.80	00:03:17	80.00%	50.00%
31.	pt-pt	**10**	1.40	00:00:23	100.00%	60.00%
32.	el	**8**	1.12	00:00:53	100.00%	87.50%
33.	es-ar	**8**	1.38	00:00:18	100.00%	75.00%
34.	hu	**6**	1.67	00:00:43	100.00%	66.67%
35.	ja-jp	**6**	1.50	00:00:26	100.00%	50.00%
36.	ko-kr	**6**	1.83	00:00:22	100.00%	50.00%
37.	pt	**6**	1.33	00:00:17	100.00%	66.67%
38.	sl	**6**	1.67	00:02:16	100.00%	66.67%
39.	ar	**5**	1.80	00:00:49	100.00%	60.00%
40.	en-ca	**5**	1.60	00:03:59	60.00%	40.00%
41.	et	**5**	1.20	00:00:26	80.00%	80.00%
42.	fi	**5**	2.00	00:00:01	100.00%	80.00%
43.	fi-fi	**5**	1.40	00:00:00	80.00%	60.00%
44.	lt	**5**	1.20	00:00:00	100.00%	80.00%
45.	pl-pl	**5**	1.20	00:04:23	80.00%	80.00%
46.	en-au	**4**	1.75	00:01:11	75.00%	50.00%
47.	es-cl	**4**	2.25	00:04:25	100.00%	50.00%
48.	sk	**4**	1.25	00:00:24	100.00%	75.00%
49.	th	**4**	1.25	00:00:29	100.00%	75.00%
50.	de-ch	**3**	1.67	00:00:44	100.00%	33.33%
51.	hu-hu	**3**	1.67	00:00:38	100.00%	33.33%
52.	lv	**3**	2.67	00:02:27	100.00%	66.67%
53.	ro	**3**	1.33	00:01:07	100.00%	66.67%
54.	zh-hk	**3**	1.33	00:00:00	100.00%	66.67%
55.	ca-es	**2**	2.50	00:04:50	100.00%	50.00%
56.	en-za	**2**	1.00	00:00:00	100.00%	100.00%
57.	fil	**2**	1.00	00:00:00	100.00%	100.00%
58.	hr	**2**	1.00	00:00:00	100.00%	100.00%
59.	nb	**2**	1.00	00:00:00	100.00%	100.00%
60.	uk	**2**	1.00	00:00:00	100.00%	100.00%
61.	vi	**2**	1.00	00:00:00	100.00%	100.00%
62.	(not set)	**1**	12.00	00:08:47	100.00%	0.00%
63.	af	**1**	1.00	00:00:00	100.00%	100.00%

64.	ar-eg	**1**	1.00	00:00:00	100.00%	100.00%
65.	ar-sa	**1**	1.00	00:00:00	100.00%	100.00%
66.	bn	**1**	1.00	00:00:00	100.00%	100.00%
67.	c	**1**	1.00	00:00:00	100.00%	100.00%
68.	cs-cz	**1**	1.00	00:00:00	100.00%	100.00%
69.	da-dk	**1**	1.00	00:00:00	100.00%	100.00%
70.	el-gr	**1**	1.00	00:00:00	100.00%	100.00%
71.	en_gb	**1**	2.00	00:00:00	100.00%	0.00%
72.	en-nz	**1**	1.00	00:00:00	100.00%	100.00%
73.	es-cr	**1**	1.00	00:00:00	100.00%	100.00%
74.	he	**1**	1.00	00:00:00	100.00%	100.00%
75.	hr-hr	**1**	3.00	00:00:28	100.00%	0.00%
76.	id	**1**	2.00	00:00:00	100.00%	0.00%
77.	is	**1**	2.00	00:00:00	100.00%	0.00%
78.	nb-no	**1**	1.00	00:00:00	100.00%	100.00%
79.	no	**1**	1.00	00:00:00	100.00%	100.00%
80.	uk-ua	**1**	1.00	00:00:00	100.00%	100.00%
81.	zh-sg	**1**	2.00	00:00:00	100.00%	0.00%

Rows 1 - 81 of 81

© 2013 Google
